# Healthcare contact days for people with stage IV non-small cell lung cancer (NSCLC) in Ontario: A population-based study

**DOI:** 10.23889/ijpds.v9i5.2490

**Published:** 2024-09-18

**Authors:** Paul Nguyen, Arjun Gupta, Christopher Booth, Timothy Hanna

**Affiliations:** 1 Queen's University; 2 University of Minnesota; 3 Queen’s Cancer Research Institute

## Background

Survival is limited with advanced NSCLC, and frequent healthcare visits can become all-consuming. We investigated patterns of contact days—days with any in-person healthcare contact as a measure of potential time toxicity—in a population-based sample.

## Methods

We created a population-based, retrospective cohort with health administrative data from Ontario, Canada, of adults with stage IV NSCLC in 2014-2017, dying 2014-2019. The primary outcome was contact days assessed from diagnosis to death. We stratified analyses by receipt, type and lines of systemic therapy administered. We plotted and fitted with cubic splines the weekly percentage of contact days to obtain trajectories over the disease course.

## Results

We identified 5,785 stage IV NSCLC patients. The median (interquartile range [IQR]) survival was 108 days (49-426), and median percentage of contact days was 33.3%. Patients receiving systemic treatment had longer median survival (261 [152-420] vs. 66 [34-130] days) and lower median percentage of contact days (22.2% vs. 40.9%). Overall and for subgroups (systemic therapy vs. not; type and lines of therapy), trajectories followed a U-shaped distribution, with highest rates immediately following diagnosis and prior to death. The difference between the maximal peak and trough was greater in patients who received systemic therapy (peak 34.8% vs. trough 15.9%, ""deeper U"") vs. not (39.5% vs. 27.6%, ""shallower U"").

## Conclusion

Stage IV NSCLC patients spent a significant proportion of days alive with healthcare contact. These data serve as a call to recognize patient time burdens, improve care efficiency, and better support patients during periods of high burden.

